# The impact of hemoadsorption in patients with chronic kidney disease undergoing coronary artery bypass grafting

**DOI:** 10.1093/ckj/sfaf241

**Published:** 2025-07-31

**Authors:** Erdal Simsek, Serdar Gunaydin

**Affiliations:** Department of Cardiovascular Surgery, University of Health Sciences, Ankara City Hospital Campus, Ankara, Turkey; Department of Cardiovascular Surgery, University of Health Sciences, Ankara City Hospital Campus, Ankara, Turkey

**Keywords:** CABG, cardiac surgery-associated acute kidney injury, CytoSorb, hemoadsorption, inflammation

## Abstract

**Background:**

With an annual incidence of up to 30%, cardiac surgery-associated acute kidney injury (CSA-AKI) may be one of the most underestimated yet common complications, hence reno-protective interventions are critical. We evaluated the impact of hemoadsorption (HA) on clinical outcomes in KDIGO (Kidney Disease: Improving Global Outcomes) G2/A2 patients (GFR 60–89 ml/min/1.73 m^2^ and 30–300 mg/g albuminuria) undergoing coronary artery bypass grafting (CABG).

**Method:**

Forty patients with chronic kidney disease (KDIGO G2/A2) were treated with intraoperative HA therapy during CABG surgery (HA group) and were compared with 40 propensity-score matched control CABG patients without intraoperative HA (control group). Primary endpoints were the need for renal replacement therapy (RRT) and/or worsening of the KDIGO stage during the perioperative period. Secondary endpoints included changes in inflammatory biomarkers, vasopressor use, and ICU/hospital stay.

**Results:**

No significant differences were observed in demographics between groups. Worsened KDIGO stages were more frequent in the control group (*P* = .04), and the HA group had less RRT use and shorter ICU stays (*P* = .02 and *P* = .03). On the first postoperative day, levels of serum creatinine (1.85 ± 0.6 vs 2.75 ± 0.6 mg/dl; *P* = .035), myoglobin (210±75 vs 310 ± 80 μg/l; *P* = .04), NT-proBNP (130 ± 30 vs 180 ± 40 pg/ml; *P* = .04), IL-6 (8.2±4 vs 22.2 ± 4 pg/ml; *P* = .012), procalcitonin (1.4 ± 0.1 vs 1.76 ± 0.2 μg/l, *P* = .02), C-reactive protein (7.6 ± 2 vs 14.2 ± 4 mg/l, *P* = .01), and D-dimer (0.76 ± 0.04 vs 2.2 ± 0.07 mg/l, *P* = .002) were significantly lower in the HA group.

**Conclusion:**

This pioneering study highlights the potential benefits of HA in mitigating kidney function and inflammation in CABG patients with borderline chronic kidney disease. These findings require validation in large, multicenter trials.

## GRAPHICAL ABSTRACT

**GRAPHICAL ABSTRACT:**
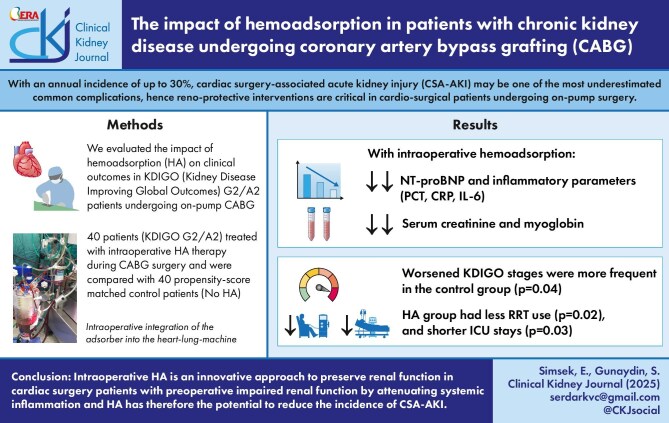

KEY LEARNING POINTS **What was known:**

- Cardiac surgery-associated acute kidney injury (CSA-AKI) has an annual incidence of up to 30%, especially in renal disease (KDIGO G2/A2).
- The inflammatory response induced by cardiopulmonary bypass plays a key role in renal injury.
- Hemoadsorption has the potential to reduce systemic inflammation.

**This study adds:**

- This propensity-score-matched study evaluated the effects of intraoperative hemoadsorption in patients with preoperative renal disease (KDIGO G2/A2) (GFR 60–89 ml/min/1.73 m^2^ and 30–300 mg/g albuminuria) undergoing CABG surgery.
- Laboratory inflammatory parameters and creatinine and myoglobin were significantly reduced by intraoperative hemoadsorption.
- Worsened KDIGO stages were more frequent in the control group (*P* = .04), and the HA group had less RRT use, and shorter ICU stays (*P* = .02 and *P* = .03).

**Potential impact:**

- The present results demonstrate that intraoperative hemoadsorption is an innovative approach to preserve renal function in cardiac surgery patients with impaired preoperative renal function.
- By attenuating systemic inflammation, this technique has the potential to reduce the incidence of CSA-AKI and improve recovery outcomes.

## INTRODUCTION

Cardiac surgery-associated acute kidney injury (CSA-AKI) is a significant perioperative complication affecting nearly one-third of patients undergoing cardiac surgery. This condition is associated with increased mortality, long-term health problems and a health economic cost burden [1]. The underlying mechanisms of CSA-AKI are complex and multifactorial, involving ischemia-reperfusion injury, oxidative stress, systemic inflammation, hemolysis, and exposure to nephrotoxic agents. The use of cardiopulmonary bypass (CPB) exacerbates these factors by triggering complement activation, hemolysis, and systemic inflammatory responses. Despite advances in understanding, significant gaps remain in elucidating the pathophysiology and individual risk factors [2].

Prevention and early detection remain the key strategies for managing CSA-AKI, as therapeutic options are often inadequate [3]. Various classification systems, such as the Risk, Injury, Failure, Loss, End-Stage, Acute Kidney Injury Network, and Kidney Disease: Improving Global Outcomes (KDIGO)/Valve Academic Research Consortium, are instrumental in categorizing patients based on risk profiles and timing of assessment for kidney injury [4].

Extracorporeal blood purification techniques, particularly hemoadsorption (HA), have emerged as promising methods to reduce systemic inflammation and improve postoperative outcomes [5].

The aim of the present study was to evaluate the efficacy of HA as an adjunctive therapy in patients with chronic kidney disease (KDIGO G2/A2: GFR 60–89 ml/min/1.73 m^2^ and 30–300 mg/g albuminuria) undergoing cardiac surgery focusing on renal, inflammatory, and clinical outcome parameters.

## MATERIALS AND METHODS

### Patient selection

A retrospective analysis of prospectively collected data was performed at a single tertiary care center within a 3-year period. Eligible participants for this retrospective study were patients aged 18 years or older with borderline chronic kidney disease (KDIGO G2/A2) scheduled for elective coronary artery bypass grafting (CABG) with an expected CPB duration of >90 minutes. The study was reviewed and approved by the institutional ethics committee (TABED 2:816).

Baseline creatinine levels obtained within 3 days before surgery were used to assess glomerular filtration rate (GFR) according to KDIGO definitions. AKI was diagnosed using KDIGO criteria based on creatinine changes (Table 1).

**Table 1:** KDIGO classification: prognosis of chronic kidney disease by GFR and albuminuria categories.

GFR (ml/min/1.73 m^2^)		
G1	normal/high	≥90
G2	mildly decreased	60–89
G3a	mild–moderate decrease	45–59
G3b	moderate–severe decrease	30–44
G4	severely decreased	15–29
G5	kidney failure	<15
Persistent albuminuria categories		
A1	normal–mildly increased	<30 mg/g
		<3 mg/mmol
A2	moderately increased	30–300 mg/g
		3–30 mg/mmol
A3	severely increased	>300 mg/g
		>30 mg/mmol

### Renal protection protocol

All patients received preoperative hydration (70 ml/h of 0.9% NaCl) and oral *N*-acetylcysteine (NAC) (600 mg twice daily) for 3 days. During CPB, pulsatile flow and ultrafiltration (1000–1500 ml) were used. This was based on the guideline published by EACTS/EACTAIC/EBCP on cardiopulmonary bypass in adult cardiac surgery; the perioperative use of intravenous NAC may be considered in patients with chronic kidney disease to reduce AKI after cardiac surgery (IIb-B) and common goals for ultrafiltration are blood concentration, filtration of unwanted substances and management of electrolyte balance [6].

### Surgical procedure

A standard median sternotomy was performed, followed by cannulation of the aorta and right atrium. Anesthesia was induced with fentanyl and pancuronium bromide, and full systemic heparinization was maintained throughout the procedure. Moderate hypothermia (32°C) was induced, and retrograde autologous priming was performed. The circuit was primed with 1200 ml (with a 200 ml safety margin in the reservoir) of Plasma-Lyte^®^ A (Eczacibasi, Istanbul, Turkey). Mean arterial pressure was maintained between 70 and 90 mmHg, and CPB flow was maintained at 2.2–2.5 l/min/m^2^. Conventional del Nido cardioplegia was used (20 ml/kg). Proximal anastomoses were performed under cross-clamp. Postoperative rewarming was initiated. CPB was discontinued when 36.5°C was reached and heparin was reversed with a dose of 3.1 mg/kg protamine sulfate after decannulation.

### Hemoadsorption

**Figure 1:**
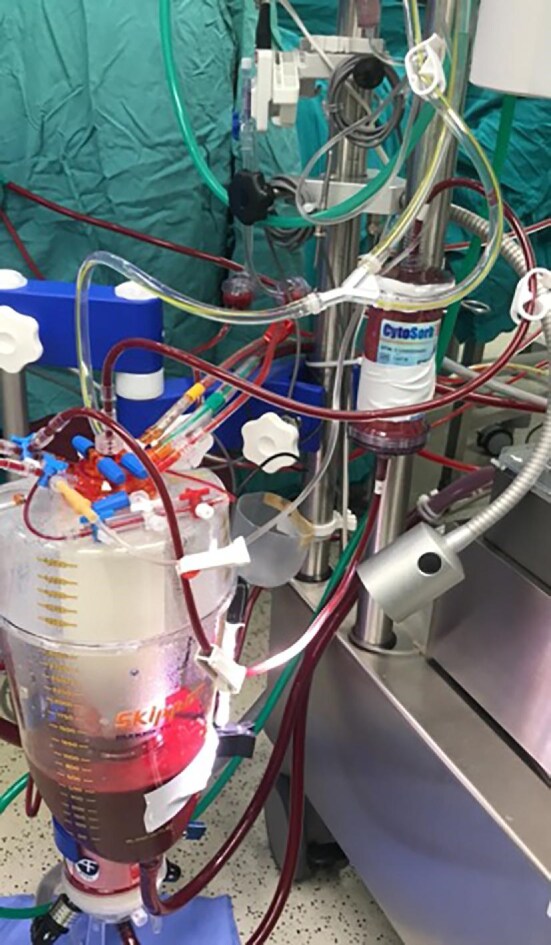
Hemoadsorber use in the CPB procedure.

The hemoadsorber (CytoSorbents, Princeton, NJ, USA) is a CE-marked medical device consisting of a highly porous, biocompatible polymer whose specific properties enable it to bind a wide range of hydrophobic compounds with a molecular weight of up to 60 kDa, a range in which most inflammatory agents, nephrotoxins, and myoglobulins are found. Removal is concentration dependent. In the study group, a 300-ml adsorber was used for the entire duration of CPB and was installed in the venous return so that blood was pumped back into the reservoir via a side arm (Fig. 1). The average flow rate through the adsorber was 500 ml/min via a separate roller pump. Each patient in the study group received continuous HA treatment intraoperatively during the pump run. Data from patients requiring further HA therapy in the ICU were excluded. Preparatory flushing of the adsorbers and heparinization during the procedures were performed according to the manufacturer's instructions [7].

### Perioperative monitoring

Complete blood count, prothrombin time, activated partial thromboplastin time, troponin I, and levels of fibrinogen, interleukin (IL) 6, C-reactive protein (CRP), procalcitonin (PCT), NT-proBNP, D-dimer, serum creatinine, GFR, and myoglobulin were recorded. Standard blood and urine chemistry results were documented. Blood samples were collected in potassium EDTA (ethylenediaminetetraacetic acid) tubes at the following intervals:

- baseline: after induction of anesthesia before CPB (T1)
- off CPB: after cessation of CPB (T2)
- ICU: first postoperative day at 8 a.m. (T3)

For each patient, the following factors were assessed and documented before discharge: hemodynamic variables, operative data, postoperative bleeding, use of blood products, incidence of arrhythmias, use of inotropic support, complications and infections, duration of ventilatory support, ICU stay and hospital stay, perioperative mortality, New York Heart Association classification, and Doppler echocardiography.

Primary endpoints were the need for renal replacement therapy (RRT)/dialysis and/or KDIGO acute kidney disease (AKD) stage in the perioperative period. KDIGO AKD stage monitoring was performed up to three months postoperatively. Secondary endpoints were changes in inflammatory biomarkers, vasopressor requirements, and ICU/hospital stay. Patients with septic shock and limited life expectancy were excluded.

### Statistical analysis

The sample size was determined based on a similar study on the impact of extracorporeal HA during prolonged CPB on the incidence of AKI [8]. Using the protocol, 36 patients in each group were evaluated as sufficient to show a statistically significant difference with 5% error and 85% power. Propensity-score matching was performed based on the following variables: age, left ventricular function, KDIGO G2/A2, preoperative serum creatinine, and GFR. Matching was performed using 1:1 nearest neighbor with a matching tolerance of 0.1 in the overall propensity score.

Data were expressed as mean ± standard deviation. Two-way analysis of variance (ANOVA) was used to analyze differences over time in each group (repeated measures ANOVA) and for differences between groups. *Post hoc* test (Bonferroni correction) was used whenever a significant difference was found. A *P* value <.05 was considered significant. Data were analyzed using IBM SPSS Statistics, version 22.0 for Windows (SPSS Inc, Chicago, IL, USA). Subsequently, either the *t*-test or the Wilcoxon or Mann–Whitney *U*-test was used for quantitative values. If the conditions for the *t*-test were met, it was used before the Wilcoxon or Mann–Whitney *U*-test.

## RESULTS

The study group included (after propensity-score matching) 40 patients treated with adjunctive intraoperative HA therapy compared with 40 control patients who did not receive the intervention. Baseline characteristics are summarized in Table 2. All patients were intermediate to high risk, with a Society of Thoracic Surgeons (STS) score of almost 8%, and all patients had impaired renal function (KDIGO grade G2/A2). There were no statistically significant differences in any of the preoperative laboratory values. Furthermore, preoperative coagulation parameters (prothrombin time, activated partial thromboplastin time, fibrinogen including D-dimer) and troponin I or NT-proBNP levels did not differ between the two groups. Notably, there were no significant differences in preoperative IL-6, PCT, or CRP levels. None of the patients from both groups received any postoperative HA.

**Table 2:** Baseline characteristics of patients.

Patients	HA group (*N* = 40)	Control group (*N* = 40)	*P*
Age (years), mean ± SD	63.2 ± 11.4	59.6 ± 10.8	>.05
Male (*n*)	22	19	>.05
BMI, mean ± SD	27.2 ± 4.5	28.1 ± 5.1	>.05
STS risk score (%), mean ± SD	7.6 ± 3.1	8.1 ± 3.8	>.05
Comorbidity (HT/DM/COPD) (*n*)	32/24/14	31/27/13	>.05
EF (%), mean ± SD	44.5 ± 8.7	40.7 ± 8.9	>.05
Creatinine (mg/dl) mean ± SD	1.43 ± 0.7	1.39 ± 0.7	>.05
GFR (ml/min/1.73 m^2^), mean ± SD	64.5 ± 8.7	61.8 ± 7	>.05
Urine albumin (mg/g), mean ± SD	148.3 ± 85	157.1 ± 90	>.05

HT, hypertension; DM, diabetes mellitus; COPD, chronic obstructive pulmonary disease; EF, ejection fraction rate.

**Figure 2:**
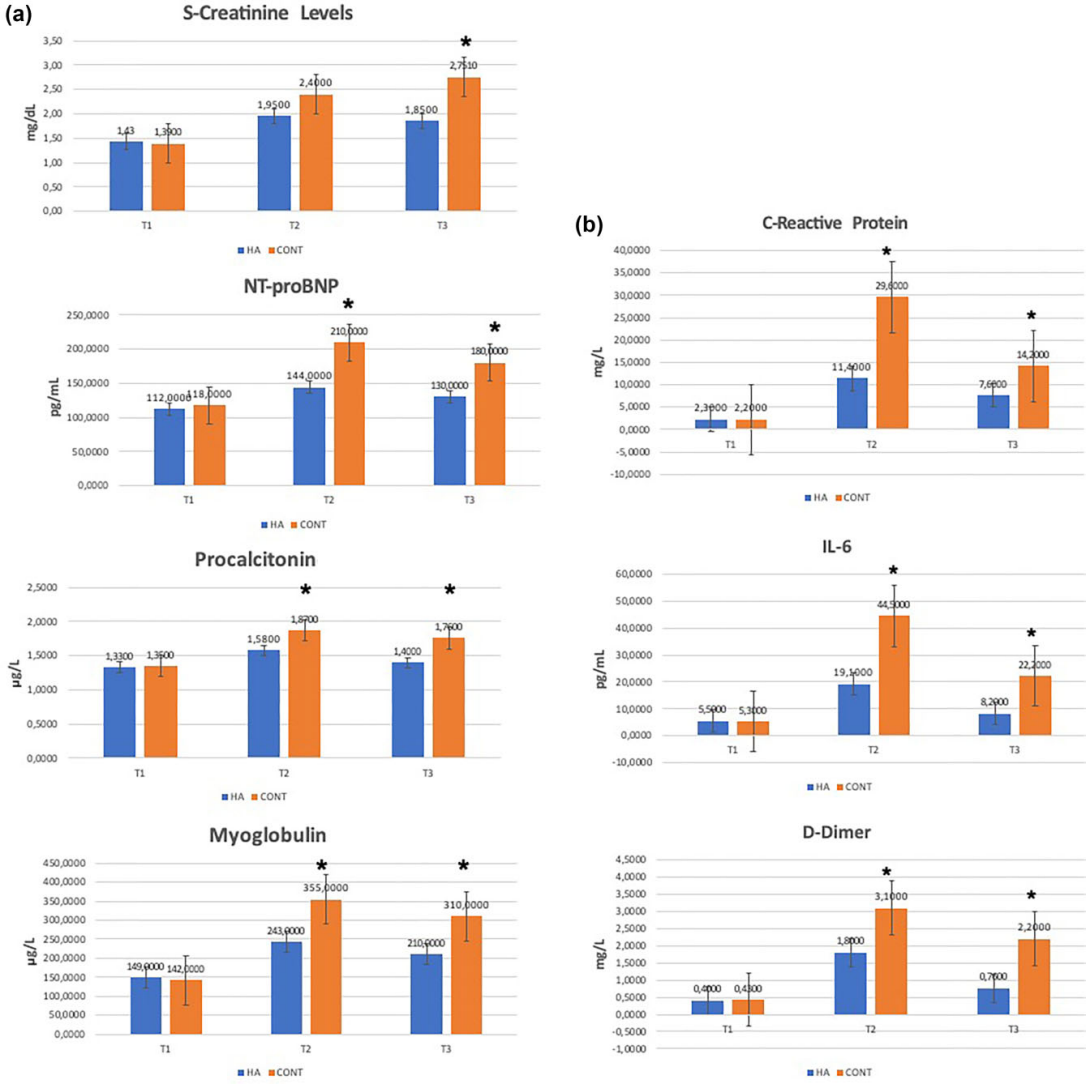
Serum creatinine and inflammatory markers (T1, baseline; T2, off CPB; T3, first postoperative day). (**a**) S-creatinine, NT-proBNP, procalcitonin, and myoglobulin. (**b**) CRP, interleukin-6, and D-dimer levels. CONT, control group. The asterisk * denotes statistically significant versus the HA study group.

Perioperative specific inflammatory markers are given in Fig. 2a and b. NT-proBNP (144±30 vs 210 ± 40 pg/ml; *P* = .03), procalcitonin (1.58 ± 0.1 vs 1.87 ± 0.2 μg/l, *P* = .03), myoglobulin (243±75 vs 355 ± 80 pg/l; *P* = .03), CRP (11.4±4 vs 29.6 ± 6 mg/l, *P* = .01), IL-6 (19.1±5 vs 44.5 ± 7 pg/l; *P* = .002), and D-dimer (1.8 ± 0.2 vs 3.1 ± 0.3 mg/l, *P* = .03) levels were significantly lower at the end of CPB (T2) in the HA group.

On the first postoperative day (T3), levels of serum creatinine (1.85 ± 0.6 vs 2.75 ± 0.6 mg/dl; *P* = .035), myoglobin (210 ± 75 vs 310 ± 80 μg/l; *P* = .04), NT-proBNP (130 ± 30 vs 180 ± 40 pg/ml; *P* = .04), IL-6 (8.2 ± 4 vs 22.2 ± 5 pg/l; *P* = .012), procalcitonin (1.4 ± 0.1 vs 1.76 ± 0.2 μg/l, *P* = .02), CRP (7.6 ± 2 vs 14.2 ± 4 mg/l, *P* = .01) and D-dimer (0.76 ± 0.4 vs 2.2 ± 0.5 mg/l, *P* = .002) were significantly lower in the HA group.

Perioperative clinical follow-up, and primary and secondary endpoints are listed in Table 3.

**Table 3:** Perioperative clinical follow-up (primary and secondary endpoints).

	HA (*n* = 40)	Control (*n* = 40)	*P*
Noradrenalin			
Need, *n*/total (%)	8 (20)	24 (60)	.02
Duration (hours), mean ± SD	19.4 ± 8	21.4 ± 9	>.05
KDIGO AKD stage	Stage 3b/A	Stage 3b/A2	.04
(3-month follow-up)	2 patients (5%)	7 patients (17.5%)	
RRT			
Need, *n*/total (%)	2 (5)	14 (35)	.02
Duration (hours), mean ± SD	28 ± 7	42 ± 10	.02
Requirement of dialysis, *n*/total (%)	0	6 (15)	.04
ICU stay (hours), mean ± SD	26.6 ± 8	58 ± 10	.03
Hospital stay (days), mean ± SD	8.2 ± 2	12.4 ± 5	.04
Mortality (3 months), *n*/total (%)	0	2 (5)	>.05

Data presented as number (%) or mean ± SD.

During their ICU stay, fewer patients in the HA group required inotropic support (8/40 vs 24/40, *P* = .02).

A statistically significant higher percentage of control patients had a worse KDIGO AKD stage (*P* = .04) in the postoperative course (3-months follow-up). This was consistent with an increased need for RRT (2/40 vs 14/40, *P* = .02) and a longer duration of RRT in the control group (28±7 vs 42 ± 10, *P* = .02), resulting in a longer ICU stay (26±8 vs 58 ± 10 hours, *P* = .03).

Of note, no complications, technical inconveniences, safety issues, or serious device related adverse events were reported with the use of the hemoadsorber.

## DISCUSSION

In the current comparative propensity-score-matched study, we sought to evaluate the effects of intraoperative HA in patients with preoperative renal disease (KDIGO G2/A2) undergoing CABG surgery. We found the following interesting results: In the postoperative course, fewer patients in the HA group showed a worsening of KDIGO status, in line with significantly lower serum creatinine levels and fewer patients on RRT. In addition, the HA group required less inotropic support during the postoperative period. Notably, inflammatory parameters were also significantly reduced in the HA group.

Patients undergoing cardiac surgery are inevitably exposed to an acute inflammatory host response triggered by both intrinsic (e.g. tissue damage, endothelial injury) and extrinsic (e.g. anesthesia, extracorporeal circulation) factors. Combined, all these factors can affect renal function by exacerbating systemic inflammatory responses, triggering complement activation or hemolysis. In cardiac surgery in particular, the inflammatory response induced by CPB plays a key role in renal injury and has been reported to lead to CSA-AKI in up to 30% of patients [9].

Recently, the detection, assessment and management of CSA-AKI in routine cardiac surgery clinical practice has remained suboptimal, especially in its early stages in preoperative borderline renal function. To prevent the progression of AKI and improve patient outcomes, there is significant room for improvement in current perioperative management strategies. Effective interventions include preoperative corticosteroid administration, leukocyte filtration, intraoperative inhaled nitric oxide and the use of KDIGO care bundles. In addition, supportive therapies such as a tailored fluid vasopressor regimen are critical [10, 11]. In our routine practice, we also prepare such patients for surgery with preoperative hydration with *N*-acetylcysteine and use pulsatile flow and goal-directed fluid management during CPB based on the latest guidelines [6].

Despite the fact that most CSA-AKI cases (approximately 90%) are classified as mild, they are ultimately associated with worse outcomes compared to control patients. Mild CSA-AKI is an independent predictor of 30-day postoperative mortality, and mortality rates increase with severity, reaching nearly 50% in the 2%–5% of cardiac surgery patients who develop severe insufficiency requiring RRT.

Intraoperative HA during CPB has shown encouraging results in several experimental and clinical studies, improving both the inflammatory profile and hemodynamic parameters in patients [12–14]. However, it is unknown whether adjunctive intraoperative HA could attenuate the inflammatory response and thereby reduce CSA-AKI in patients with borderline renal disease. Recently, two RCTs have been published showing improved renal function in patients undergoing cardiac surgery: The RECCAS (Removal of Cytokines during Cardiac Surgery) trial evaluated the use of HA in patients with prolonged CPB duration (>90 minutes)(15). Although Hohn et al. were unable to demonstrate significant differences in inflammatory parameters, the RECCAS study showed secondary benefits, such as reduced duration of RRT (thus improving renal recovery) and also improved hemodynamic stability in the postoperative period. The authors reported a significantly shorter duration of RRT in the HA group (2.3 ± 0.6 days) compared to the control group (5.3 ± 1.2 days; *P* = .029). Taken together, these results highlight the potential of HA to improve renal and systemic outcomes in patients with AKI following cardiac surgery, rather than focusing solely on cytokine removal as demonstrated by Jansen's experiments [11, 15, 16]. The second recently published RCT, SIRAKI02, evaluated the oXiris^®^ filter and found no reduction in IL-6. However, in line with RECCAS, the authors also demonstrated a significant reduction in AKI incidence from 39.7% in the control group to 28.4% in the intervention group (*P* = .03). They could also identify the following factors to be associated with CSA-AKI: advanced age, female gender, preoperative chronic kidney disease, decreased left ventricular function, and longer CPB duration during surgery [13].

Although the two previously mentioned trials and our analysis focused on the intraoperative application of HA to prevent CS-AKI, there are some differences between these trials and the present analysis. The SIRAKI02 trial used the oXiris filter and the primary aim was to evaluate CSA-AKI in high-risk patients undergoing cardiac surgery. The RECCAS trial aimed to evaluate the impact of HA using CytoSorb on postoperative systemic inflammatory response. In contrast to the SIRAKI02 and RECCAS trial, the aim of the present analysis was to investigate whether CSA-AKI could be prevented by intraoperative HA in patients with impaired preoperative renal function. Moreover, the current trial combined clinical endpoint parameters with inflammatory parameters. The observed reduction of inflammatory parameters in the HA group might be the underlying mechanism of renal protection. Of note, the idea that HA might have a positive impact on CSA-AKI is rather new and innovative and needs further investigation in larger future trials.

In this analysis, we clearly demonstrated significantly lower levels of creatinine and key inflammatory markers such as procalcitonin, myoglobin, CRP, IL-6, but also NT-proBNP and D-dimer in the HA group. Most importantly, we were able to demonstrate similar results to RECCAS and SIRAKI02 in terms of secondary clinical outcomes [13, 15]. Overall, in the present analysis, worse KDIGO AKD stages were more frequent in the control group (*P* = .04), and the HA group had less RRT use and a mean CPB duration of >100 minutes, comparable to the inclusion criteria of RECCAS. The effect of HA in removing harmful pathogens may therefore offer reno-protective effects.

Moreover, a recent study showed that the prevalence of CSA-AKI was high and directly related to the preoperative risk profile of patients [17]. The patients included into the present study also had a high-risk profile with a STS score ∼8%.

In the present analysis, only intraoperative HA was investigated. Future studies should also include postoperative continuation of the therapy which might add further benefit. This could be achieved by an innovative approach using a hemadsorption cartridge integrated into a stand-alone hemoperfusion pump.

Another important finding was the significant reduction in myoglobin in the current analysis. Once released in high concentrations, myoglobin induces damage to the proximal tubules via reactive oxygen species, reducing excretory function, while myoglobin precipitates block the distal tubules, which may also be the case in CSA-AKI and a potential additional cause of the high incidence of AKI during cardiac surgery. The rapid removal of myoglobin and the attenuation of inflammation, both of which contribute to the development of AKI, is the therapeutic goal and the rationale for the use of HA.

Our study has some important limitations that need to be considered when interpreting the results. First, although we performed a power analysis, our patient population can still be considered small. A higher number of cases would change the borderline statistical differences such as inotrope use, hospital stay, and mortality. Second, the lack of blinding during surgery may have influenced intraoperative decisions such as fluid administration or use of ultrafiltration. Perioperative fluid management strategies may also have confounded serum creatinine measurements. Finally, both groups were comparable after propensity-score matching, but bias cannot be completely excluded.

## CONCLUSION

In conclusion, the present results demonstrate that intraoperative HA using is an innovative approach to preserve renal function in cardiac surgery patients with preoperative borderline renal function. By attenuating systemic inflammation, this technique has the potential to reduce the incidence of CSA-AKI and improve recovery outcomes. Further investigation is warranted to refine the methods of HA application and to assess its long-term clinical benefits.

## Data Availability

The data underlying this article are available in the article.

## Notes

Presented at the 61st European Renal Association Congress, 23–26 May 2024, Stockholm, Sweden
